# *BMC Pharmacology and Toxicology* reviewer acknowledgement 2015

**DOI:** 10.1186/s40360-016-0049-x

**Published:** 2016-02-17

**Authors:** Christopher Morrey

**Affiliations:** BioMed Central, Floor 6, 236 Gray′s Inn Road, London, WC1X 8HB United Kingdom

## Abstract

The editors of *BMC Pharmacology and Toxicology* would like to thank all our reviewers who have contributed to the journal in Volume 16 (2015).

**Lamya Alnaim**

Saudi Arabia

**Guzmán Alvarez**

Uruguay

**Solomon Amade**

Ethiopia

**Richard Amison**

UK

**Salvatore Amoroso**

Italy

**Karl-Erik Andersson**

USA

**Paulo Arnaldo**

Mozambique

**Oscar Arrieta**

Mexico

**Alfred Balch**

USA

**Douglas Ball**

Philippines

**Jo Barnes**

New Zealand

**Perihan Başhan**

Turkey

**Frank Beier**

Canada

**Raja Benkirane**

Morocco

**Amanda Bess**

USA

**Jiraganya Bhongsatiern**

USA

**Margherita Brindisi**

Italy

**Stephen Britland**

UK

**Simone Brogi**

Italy

**Florent Busi**

France

**Rainer W Bussmann**

USA

**Gillian Butler-Browne**

France

**Sabrina Calil-Elias**

Brazil

**Sarah Campbell**

USA

**Domenico Capone**

Italy

**Mariella Carrieri**

Italy

**Mauro Cataldi**

Italy

**Mauricio Chagas Da Silva**

Canada

**Kathryn Chapman**

UK

**Sara Cheleschi**

Italy

**Yingjia Chen**

USA

**Imti Choonara**

UK

**Lubica Cibickova**

Czech Republic

**Bart Crielaard**

Netherlands

**Elena Crisa**

Italy

**Anna Czlonkowska**

Poland

**Nissar Darmani**

USA

**Fabrizio De Ponti**

Italy

**Saskia De Wildt**

Netherlands

**Monorama Deb**

India

**Chris Delcher**

USA

**Domenico Delfino**

Italy

**John Dempster**

UK

**Antonello Di Paolo**

Italy

**Kawa Dizaye**

Iraq

**Matthew Drake**

USA

**Thomas Efferth**

Germany

**Damon Eisen**

Australia

**Omar Elmi**

Malaysia

**Suleiman El-Sharif**

United Arab Emirates

**Eric Engleman**

USA

**Elena Enioutina**

USA

**Sebnem Eren Cevik**

Turkey

**Yu Fang**

China

**Wissam Faour**

Lebanon

**Qiping Feng**

USA

**Peter Ferenci**

Austria

**Carmela Fimognari**

Italy

**Carmine Finelli**

Italy

**Andrea Fiorillo**

Italy

**Sheryl Flanagan**

Canada

**Samuel Fountain**

UK

**Dana García**

USA

**Stephen Gbedema**

Ghana

**Yubin Ge**

USA

**Giorgio Gentile**

Italy

**Saeid Ghavami**

Canada

**Alessandra Gianoncelli**

Italy

**Jean-Pierre Gillet**

Belgium

**Ioannis Gioulbasanis**

Greece

**Danijela Gnjidic**

Australia

**Helen Griffiths**

UK

**Tai Guo**

USA

**Manjeeta Gupta**

India

**Peter Hansell**

Sweden

**Mohamed Hassan**

USA

**Medardo Hernández**

Spain

**Mohammad Hojjat-Farsangi**

Sweden

**Joseph Horzempa**

USA

**Tzu-Bou Hsieh**

Canada

**Yi-Shin Huang**

Taiwan

**Risto Huupponen**

Finland

**Masahiro Ikeda**

Japan

**Masahide Ikeguchi**

Japan

**Manish Issar**

USA

**Shazia Jamshed**

Malaysia

**Tong-Rong Jan**

Taiwan

**Sophia Johnson**

USA

**Ebru Karpuzoglu**

USA

**Imit Kaur**

USA

**Ron Keizer**

Sweden

**Ulrike Kemmerling**

Chile

**Martin Kimmel**

Germany

**Eugene Kiyatkin**

USA

**Curtis Klaassen**

USA

**Jos Kosterink**

Netherlands

**Sperry Kotsianas**

USA

**Detlef Krieter**

Germany

**Laura Kubik**

USA

**Subramaniansenthil Kumaran**

India

**Chun Shing Kwok**

UK

**Mathew Yamoah Kyei**

Ghana

**Malcolm Lader**

UK

**Vincenzo Lariccia**

Italy

**Cecily Rosemary Latha**

India

**John Lavelle**

USA

**Dario Lehoux**

Canada

**Shing-Jong Lin**

Taiwan

**Duan Liu**

USA

**Kai Liu**

USA

**Xiaoxi Liu**

USA

**Tingting Liu**

USA

**Jie Liu**

USA

**Vincenza Rita Lo Vasco**

Italy

**Diomedes Logothetis**

USA

**Bal Lokeshwar**

USA

**Yi Lu**

USA

**Francesca Luchetti**

Italy

**Simona Magi**

Italy

**Anke Hilse Maitland-Van Der Zee**

Netherlands

**Francois Marceau**

Canada

**Zoratti Mario**

Italy

**Timothy Martland**

UK

**Wojte Marusza**

Poland

**María-Victoria Mateos**

Spain

**Isao Matsui-Yuasa**

Japan

**Lynn McPherson**

USA

**Bruce Melancon**

USA

**Yongfan Men**

USA

**Torri Metz**

Colombia

**Tim Millar**

UK

**Seigo Minami**

Japan

**Tomoya Mita**

Japan

**Maria Miteva**

France

**Julio Cesar Morales-Medina**

Mexico

**Wondemagegn Mulu**

Ethiopia

**Karl Nocka**

USA

**Lucy Okell**

UK

**Olufunmiso Olusola Olajuyigbe**

South Africa

**Pierluigi Paggiaro**

Italy

**Frank Paloucek**

USA

**Manlio Palumbo**

Italy

**Hitesh Pandya**

UK

**Nazareno Paolocci**

USA

**Roberto Passera**

Italy

**Davide Pastorelli**

Italy

**Aarti Patel**

New Zealand

**Vidya Perera**

USA

**Elisabetta Poluzzi**

Italy

**Carol Poskay**

USA

**Casey Quinlan**

USA

**Rapur Ram**

India

**Rubhana Raqib**

Bangladesh

**Balmiki Ray**

USA

**Jerrie Refuerzo**

USA

**Carlo Reggiani**

Italy

**Roberto Rimondini**

Italy

**Anka Roehr**

Germany

**Joseph Rower**

USA

**Stephen Safrany**

UK

**Anke Schiedel**

Germany

**Verena Schneider-Lindner**

Germany

**Yutaka Seino**

Japan

**Abhishek Sharma**

USA

**Filomena Silva**

Portugal

**Anurag Singh**

USA

**Ken Soderstrom**

USA

**Jean Tafforeau**

Belgium

**Yoshikazu Takaesu**

Japan

**Giovanni Tarantino**

Italy

**Anthony Taylor**

UK

**Cheong Teng**

Malaysia

**Manish Tiwari**

USA

**Sarah Tonkin-Crine**

UK

**Anna Toso**

Italy

**Pramote Tragulpiankit**

Thailand

**Nam Tran**

USA

**Arnaud Trébucq**

France

**Gianluca Trifirò**

Italy

**Fulvio Ursini**

Italy

**Johannes Van Der Wouden**

Netherlands

**Benjamin Van Tassell**

USA

**Sarah Vecchi**

Italy

**Raja Venkatasubramanian**

USA

**Margreee Vissers**

New Zealand

**Ju Wang**

USA

**Jeffrey Wang**

USA

**Yunbiao Wang**

China

**Yibo Wang**

Canada

**Phurpa Wangchuk**

Australia

**Dereje Woldemichael**

Ethiopia

**Joella Xu**

USA

**Josef Yayan**

Germany

**Venkata Yellepeddi**

USA

**Frederique Yiannikouris**

USA

**Ebru Yildirim**

Turkey

**Tian Yu**

USA

**Jiabin Zhang**

USA

**Weixia Zhong**

USA

**Meijia Zhou**

USA

**Michele Zoli**

Italy

